# Feasibility, acceptability and potential efficacy of a virtual physical activity program in primary and secondary schools in New South Wales, Australia: A quasi‐experimental study

**DOI:** 10.1002/hpja.662

**Published:** 2022-09-23

**Authors:** Kayla Elliott, Jennifer Norman, Karen Wardle, Pip Budgen, Hayley Callahan, Michelle Camilleri, Alannah Romeo, Katie Trinh, Anthony Okely, Katharina E. Kariippanon

**Affiliations:** ^1^ Early Start, School of Health and Society University of Wollongong Wollongong New South Wales Australia; ^2^ Illawarra Health and Medical Research Institute University of Wollongong Wollongong New South Wales Australia; ^3^ Health Promotion Service Illawarra Shoalhaven Local Health District, NSW Health Warrawong New South Wales Australia; ^4^ School of Health and Society University of Wollongong Wollongong New South Wales Australia; ^5^ Health Promotion Service, South Western Sydney Local Health District NSW Government Department of Health Liverpool Australia; ^6^ Health Promotion Branch, Wellbeing SA Adelaide South Australia Australia

**Keywords:** cardiorespiratory fitness, implementation science, physical activity, primary schools, school health promotion, secondary schools

## Abstract

**Issue Addressed:**

Child and youth participation in physical activity (PA) is fundamental for healthy development and obesity prevention. Government policy requires schools to offer 150 minutes of PA each week, however compliance is low. Race around Australia (RAA) is a New South Wales (NSW) Department of Education, virtual PA program aimed at assisting schools in meeting the PA guidelines.

**Methods:**

A pre‐ and post‐intervention, quasi‐experimental study was conducted using a mixed‐methods approach comprising teacher interviews, a student questionnaire and a 1.6 kilometre (km) timed run. Data were collected from April to September 2021 among students and teachers in Grades 5 to 8, from 10 schools in NSW, Australia.

**Results:**

The analytical sample included data from 918 students and 17 teachers. The RAA program was deemed feasible and acceptable in primary schools, whereas there were several systemic and intrapersonal barriers to implementation success for secondary schools. In primary schools, RAA increased PA opportunities and the 1.6 km timed runs revealed a statistically significant treatment by time effect in favour of the intervention group for cardiorespiratory fitness (−36.91 seconds, 95% CI [−63.14, −10.68], *P* = .006).

**Conclusions:**

RAA has demonstrated feasibility and potential efficacy in improving cardiorespiratory fitness. We recommend that program refinement be made to deliver an intervention that addresses the unique barriers of the secondary school setting through a multi‐level ecological approach.

**So What?:**

Despite evident benefits, implementation of PA initiatives in the school setting reveals many challenges. Stronger consideration of the Health Promotion with Schools Framework is evidently needed.

## 1 INTRODUCTION

In Australia, almost one‐quarter (24.9%) of children and young people aged 5 to 17 years are overweight or obese,^1^ whilst in the state of New South Wales (NSW), 19.3% of children are affected.^2^ Sufficient levels of PA are key for obesity prevention.^3^ Appropriate PA levels in children and young people are beneficial for the development of cardiorespiratory and muscular fitness, and healthy weight^4^, ^5^, ^6^; are positively associated with motor skill development, bone health and reduced psychological stress; enhance wellbeing, cognitive performance, and classroom behaviour and are predictive of overall health.^4^, ^6^ These associations are strengthened by participation in moderate‐to vigorous‐intensity PA (MVPA).^6^

Insufficient PA levels in children and young people are a major risk factor for obesity, chronic disease and health problems into adulthood.^4^, ^7^ An increasing evidence base recognises youth sedentary behaviour as an independent risk factor for poor physical, social and mental health.^8^ Specifically, higher levels of sedentary behaviour have been positively associated with cardio‐metabolic risk factors, unfavourable body composition, low fitness, poor behavioural conduct and low self‐esteem.^8^, ^9^

Schools have been identified as a key setting for increasing PA levels and decreasing sedentary levels,^2^, ^3^, ^10^, ^11^ given children and young people spend approximately 35 hours a week attending school.^2^ Schools present an opportunity to reach a range of students, regardless of ethnicity, gender or socio‐economic status.^10^, ^11^ The NSW Department of Education (DoE) Sport and PA Policy outlines the requirement that schools offer students opportunities to participate in a minimum of 150 minutes of planned, moderate PA with some vigorous PA each week.^12^ Data indicate that compliance with the policy is low, with only 66% of primary school students and 38% to 45% of Grade 8 to 10 secondary school students receiving at least 120 minutes of physical education each week.^13^

Classroom movement integration aims to increase PA and decrease sedentary time beyond school physical education lessons, by integrating movement into scheduled classroom time.^11^ Systematic reviews have demonstrated positive outcomes in relation to participation in PA,^14^, ^15^, ^16^, ^17^ enjoyment in PA^16^ and school‐related outcomes such as classroom behaviour^14^, ^15^, ^17^ and academic performance.^16^ The literature predominantly focuses on the primary school setting^14^, ^15^, ^16^, ^17^ with few studies reporting on secondary schools, and few studies reporting cardiorespiratory fitness outcomes of active‐break interventions. Evidence suggests that cardiorespiratory fitness is a proxy indicator for PA outcomes in children and youth,^18^ and could lead to a more accurate representation of intervention success than focusing solely on PA markers.^19^

Further research is needed to understand the outcomes associated with classroom‐based movement integration programs.^16^, ^17^, ^20^, ^21^ Additionally, research specific to the adoption and maintenance phases of implementation is required to identify facilitators for implementing school‐based PA interventions in real‐world conditions, at scale.^21^, ^22^

### 1.1 The race around Australia program

As part of their mandate, the DoE School Sport Unit are required to provide advice and assist in developing supporting materials to facilitate the implementation of the Sport and PA Policy in NSW schools.^12^ Race around Australia (RAA) is a School Sport Unit PA initiative that is part of a suite of interventions that make up the NSW Premier's Sporting Challenge.^23^ RAA was developed by a Grade 10 secondary school teacher, it was piloted across 46 NSW DoE schools in 2020 and in Term 2 2021, was formally offered to all 2220 NSW DoE schools. Once registered, students complete short bursts of PA during class time, utilising the RAA workouts, convert these activities to kilometres (kms) and enter this distance on the RAA online platform (Figure S1). The distance is then displayed on a map, tracking students, classes and schools as they race a set course around the coast of Australia over the course of a term. As students progress around the map (Figure S2), they unlock stage‐specific curriculum blogs.^24^

### 1.2 Present evaluation

The previous pilot evaluation of RAA across 46 schools, reported positive results for the program through the analysis of self‐reflection and feedback from staff and students, an evaluation of teacher and student engagement in the online platform, semi‐structured interviews and website analytics. However, the evaluation did not objectively measure any health indicators associated with the intervention.^24^ Consequently, our evaluation aimed to investigate the potential efficacy of RAA by including an assessment of cardiorespiratory fitness. A secondary aim was to examine the feasibility and acceptability of the upscaled version of RAA across primary and secondary school settings, by exploring barriers and facilitators to implementation.

## 2 METHODS

### 2.1 Study design

A pre and post intervention, quasi‐experimental study was conducted using a between‐subject design and mixed methods approach.^25^ Ethical approval was obtained from the University of Wollongong Human Research Ethics Committee (Reference 2021/ETH00065) and the NSW State Education Research Applications Process. Informed written consent was obtained from all school principals and individual teachers involved in the focus groups. An opt‐out approach was utilised for student recruitment, with study information distributed to parent/caregivers via each school's preferred forum. Opt‐out consent was deemed the most appropriate approach for students in this study due to the low risk associated with the research and the need to recruit a representative sample. This study aligns with all opt‐out consent requirements as outlined in Section 2.3 within the National Statement on Ethical Conduct in Human Research.^26^ Additionally, student assent was sought prior to data collection.

### 2.2 Participants

All DoE primary and secondary schools within the Illawarra Shoalhaven and South Western Sydney Local Health Districts were invited to participate in the evaluation. We aimed to recruit three control and three intervention schools within each health district, restricting data collection to children in Grades 5 to 8. By selecting these age groups, analyses would capture the transition from primary to secondary school and highlight any differences between the two settings. Additionally, the tools utilised in this study have been validated for these age groups.^18^

Intervention schools were recruited from schools that had registered an interest in implementing RAA; conversely control schools were recruited from those who had not registered interest in RAA. We aimed to match intervention and control schools across a range of variables, including school size, geographic location, Indices for Socio‐Economic Disadvantage, and the Indices of Community Socio‐Educational Advantage, to ensure compatibility.

All schools who participated in the evaluation received $1000 to be spent on sporting equipment, as an acknowledgement of their efforts, presented at the conclusion of data collection.

### 2.3 Intervention

Intervention schools registered in the RAA challenge and, with support from the School Sport Unit, implemented the program autonomously for 9 weeks. Control schools continued with their regular activities throughout the term.

### 2.4 Theoretical framework

Two key frameworks commonly utilised in children's health promotion, formed the theoretical basis of the analysis. ‘*Health Promotion with Schools: A Policy for The Health System*’^27^ is a NSW Health policy framework developed on the foundations of the World Health Organization's ‘*Global Standard for Health Promoting Schools*’ and acknowledges partnerships between health and education sectors as key to effective health promotion.^27^ The framework emphasises consideration of the curriculum, community and school environment as essential for successful implementation of health programs in schools.^27^

**FIGURE 1:**
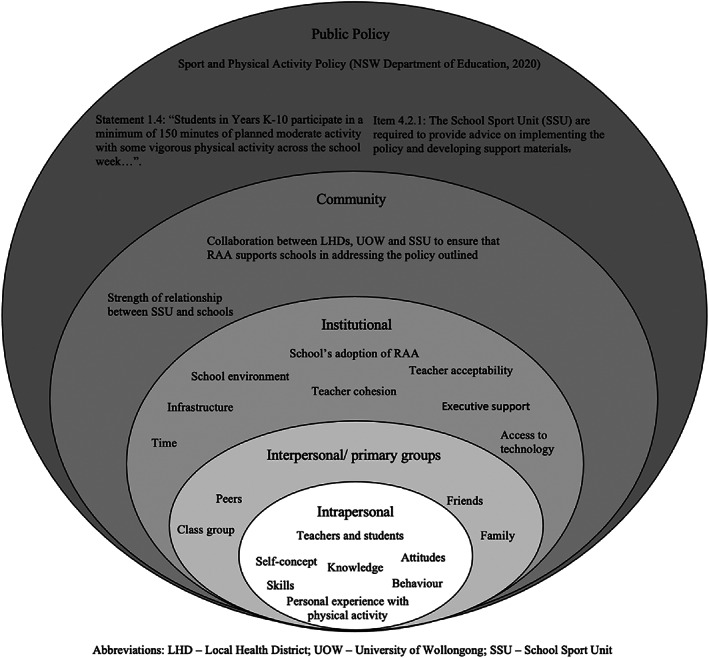
The levels of the socio‐ecological model of health in relation to race around Australia, adapted from Mcleroy et al^28^

Programs that target PA determinants at the various levels of the ecological model,^28^ hold the greatest potential for preventing and reducing obesity levels.^29^ Therefore, the study design and evaluation tools examined PA determinants related to RAA across the institutional, interpersonal and intrapersonal levels of the ecological model (Figure 1).

### 2.5 Outcome measures

The quantitative and qualitative tools utilised to assess the outcomes are detailed in Table 1.

**TABLE 1:** Outcome measures and tools used in this evaluation

Outcome	Outcome measure	Data collection tool
Acceptability	Student acceptance and enjoyment Teacher acceptance and enjoyment Likelihood of future participation	Student survey – acceptability questions Semi‐structured interviews/focus groups with teachers
Feasibility	Barriers and facilitators to implementation and participation	Semi‐structured interviews/focus groups with teachers
Efficacy of RAA in improving students' PA levels	Perceived changes in levels of PA	Semi‐structured interviews/focus groups with teachers
Efficacy of RAA in improving students' cardiorespiratory fitness	Change in time taken to complete the set course	1.6 km fitness test

Schools had the option to complete all surveys either online via Qualtrics (Qualtrics, Provo, UT), or in hardcopy form. Demographic variables including age, gender, Indigenous status and country of birth, were collected via student surveys at baseline. Acceptability data collected from intervention schools at follow up, asked students on a 5‐point Likert scale (1 = Strongly Disagree to 5 = Strongly Agree), to rate their experiences and feelings about participating in RAA. In most schools, surveys were administered to students by their teachers. Student cardiorespiratory fitness was measured at baseline and follow up via a 1.6 km fitness test conducted within each school by the research team. All teaching staff who participated in the delivery of RAA were invited to attend school level focus groups to evaluate the feasibility and acceptability of the initiative. Due to COVID‐19 lockdowns, these were conducted via videoconference using Zoom (Zoom Video Communications Inc.). A semi‐structured interview guide was developed, asking open‐ended questions, to ensure that key barriers or facilitators unknown to the interviewer were captured.^30^ The interviews were recorded and later transcribed using transcription software (Otter.ai).

### 2.6 Data analysis

#### 2.6.1 Quantitative analysis

Data were analysed in SPSS for Windows version 25 (Armonk, NY: IBM Corp). Demographic data were analysed using descriptive frequencies. Chi‐square tests of independence were used to compare acceptability responses from the primary and secondary students for each variable.

Due to an imbalance in pre and post data collection numbers for the fitness measures, data were imputed utilising the Baseline Observation Carried Forward method^31^ and analysed separately by school type. Linear mixed models were run to determine changes in the outcome measures between time points and treatment groups, with school name included as a random effect in the model to account for clustering. Differences between treatment types were considered statistically significant at *P* < .05. Effect sizes (Cohen's *d*) were calculated by dividing the difference between the means by the pooled standard deviation.^32^ To date, there is no known software that produces effect size from a linear mixed model, so these were calculated using the unadjusted means.

### 2.7 Qualitative analysis

Data were analysed following the guidelines for thematic analysis outlined by Braun and Clarke.^33^ Following familiarisation with the data, each transcript was analysed using an inductive, open coding process, whereby meaningful quotes or key examples from teachers, were assigned a code. These codes were then grouped together to develop themes. Review of the themes by the research team helped attenuate individual bias from the analysis and add credibility to the findings.

## 3 FINDINGS AND DISCUSSION

The findings are presented together with a discussion of relevant literature as suggested by Blignault and Ritchie.^34^ Recruitment occurred during March 2021. Six intervention schools and four control schools participated in the evaluation: five schools from each health district and an equal number of primary and secondary schools in total. Data were collected from April to September 2021. Given intervention schools signed up to RAA prior to recruitment for this study, principals and/or teachers at these schools are likely to be more supportive of school‐based PA when compared to those schools that had not registered to participate and were therefore eligible as control schools. This potential bias is important to consider across both the quantitative and qualitative findings.

### 3.1 The sample

**FIGURE 2:**
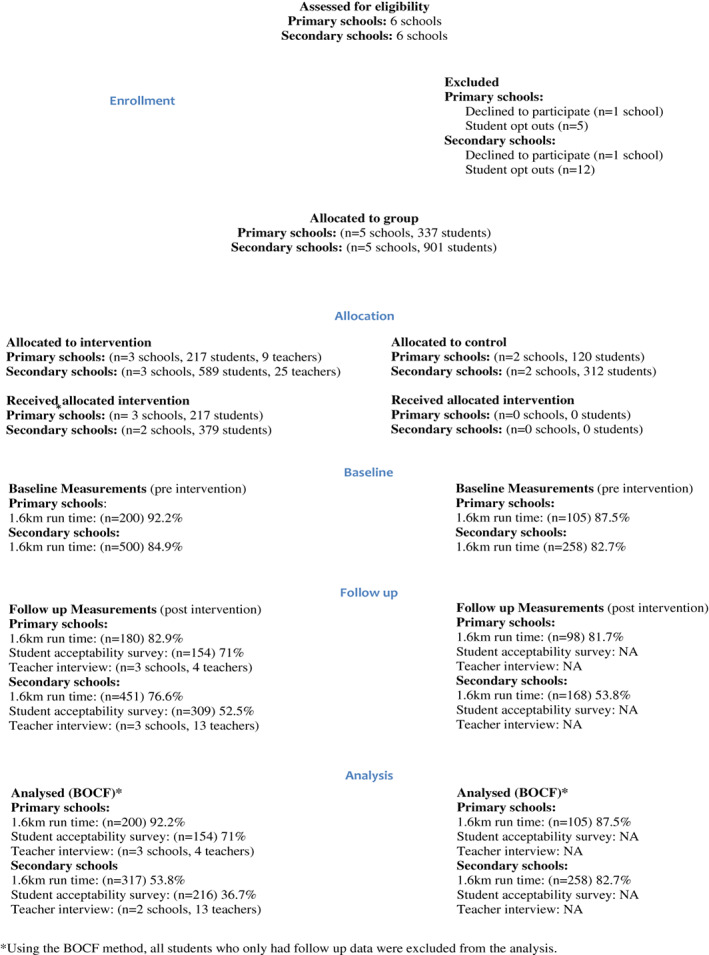
CONSORT flow chart showing the progression of participants through the study

Figure 2 illustrates the flow of participants from baseline to follow up, according to CONSORT reporting guidelines. Five eligible primary school students opted out of the evaluation. Of the five primary schools and 337 students enrolled in the study, 90.5% were included in the analytical sample for cardiorespiratory fitness. Twelve eligible secondary school students opted out of the evaluation, leaving five schools and 901 secondary school students enrolled in the study. Interviews with teachers revealed that one of the intervention secondary schools had not implemented the program as intended, hence the decision was made by the research team to exclude this school's data from the quantitative analysis. Therefore, of the 901 secondary students enrolled, 63.8% were included in the analytical sample for cardiorespiratory fitness.

Demographic descriptors including gender, Indigenous status and country of birth were similar across intervention and control groups. The mean age of primary school students in the intervention group was 10.6 years (SD = 0.68) and control group 10.3 years (SD = 1.55). The mean age for secondary students was 11.9 years (SD = 0.47) in the intervention group and 12.0 years (SD = 0.46) in the control group. There was a statistically significant difference of 1.5 months between treatment groups in secondary schools, however, the implications for this current study were deemed not meaningful. There were no statistically significant differences in gender between intervention and control groups.

Six focus groups/interviews were conducted involving all intervention schools, with feedback from four primary and thirteen secondary school teachers and RAA Coordinators (Figure 2). The qualitative analysis included the secondary school data that were excluded from the quantitative analysis, as their feedback was useful in understanding the barriers that contributed to the lack of program fidelity.

### 3.2 The feasibility and acceptability of RAA

**FIGURE 3:**
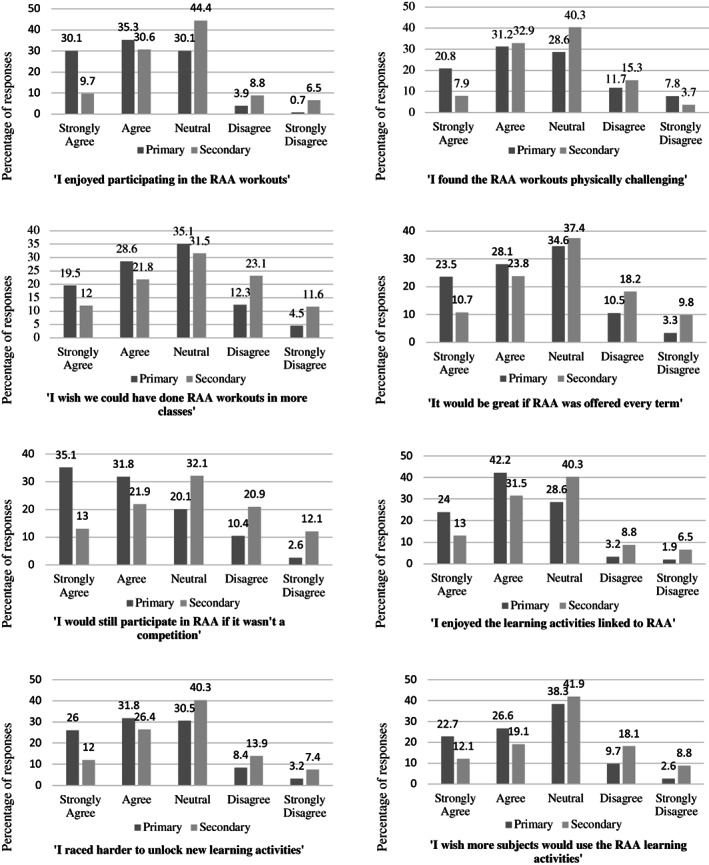
Primary and secondary students' Likert scale responses to the statements pertaining to the acceptability of RAA

Figure 3 shows the responses of primary and secondary school students to the survey questions pertaining to the acceptability of RAA. Ensuring PA is enjoyable and highlighting the positive social aspects for children is crucial for their engagement in PA interventions.^29^ Primary students' responses to questions relating to enjoyment and future participation in RAA were more positively skewed when compared to secondary students (*P* < .01) with the mode and median responses of secondary school students ‘neutral’ for each question. Teacher interviews provided feedback on student enjoyment. One primary school teacher stated:

‘*… to just be able to do a fitness activity as a brain break was great… the kids really enjoyed it*.’ – Primary teacher

Secondary school teachers provided varying feedback on student enjoyment. One teacher stated:

‘*It took a while for the kids to get into it. But overall, I think it was effective. They got interested to see about travelling around Australia…that gave them something to look forward to*.’ – Secondary teacher

Comparatively, a RAA secondary school coordinator spoke to their challenges in engaging students.

‘*And the Year 8 group that I had, became disengaged. So it's a bit hard when the kids aren't willing participants, they're not as eager as they are when they're younger, unfortunately*.’ – Secondary RAA Coordinator

The level of enjoyment aligns with previous findings of movement integration interventions in primary schools,^21^, ^35^, ^36^ however contrasts with Leahy and colleagues^37^ who found an overall positive level of satisfaction and enjoyment amongst Year 11 participants of ‘Burn2Learn’. Few other studies have explored this theme in secondary schools, however a significant, decreasing trend of engagement in PA and in‐school PA from childhood to adolescence has been reported.^38^

Despite strong engagement with the ‘race’ element of RAA, 66.9% of primary school students strongly agreed or agreed with the statement ‘I would still participate in RAA if it wasn't a competition’ in comparison with secondary students (34.9%). Overall primary school students demonstrated a positive affect toward the learning activities, compared to mode responses of ‘neutral’ from secondary school students. However, the mode and median responses for both primary and secondary school students for the statement ‘I wish more subjects would use the RAA learning activities’ was neutral.

The interviews and focus groups facilitated rich discussion and feedback, shedding light on the unique barriers and facilitators to implementation across the two distinct settings. The discussion allowed us to unpack the implications of these barriers and facilitators on the acceptability and feasibility of RAA and provided contextual detail that assisted with interpreting the quantitative results. Three key themes, relevant to both primary and secondary school contexts, were derived: school approach, teacher development and resources and engagement with the RAA program design elements. These three themes and corresponding sub‐themes are displayed in Table 2.

**TABLE 2:** Key findings from interviews with primary and secondary intervention school teachers

Theme	Sub‐themes	Explanation
School approach	Principal and school executive support Teacher collaboration Cross curricular adoption	Each school's adoption of RAA, whether that be a whole‐school approach or teacher led approach, and how that impacted implementation.
Teacher development and resources	Perceived benefits Workout resources Confidence	The skills, attitudes and beliefs of teachers, how this impacted acceptability, and the role training and resources could play in addressing intrapersonal barriers.
Engagement with the RAA program design elements	Curriculum blogs The ‘race’ element Student leadership Class cohesion	The extent to which the ‘race’ aspect of RAA increased engagement and created benefits for students and teachers.

### 3.3 Theme 1: School approach

The strength of each school's approach was a key predictor for the feasibility and acceptability of RAA. This included the level of principal or school executive support, teacher collaboration and cross‐curricular adoption.

#### 3.3.1 Principal and school executive support

In both primary and secondary schools, teachers perceived their principals and school executives as supportive of the implementation of RAA, however uninvolved in the processes of recruiting teachers and ensuring ongoing implementation of the program. Previous research has noted factors relating to the ‘delivery system’, including administrative/managerial support, and the availability of resources, time and space, as key predictors of the adoption, implementation and sustainability of school‐based PA interventions.^21^, ^22^, ^36^, ^39^, ^40^

‘*All executives were really supportive… from a friendly distance, if that makes sense? They kind of let us go with the flow and learn and do as we're doing*.’ – Primary teacher

### 3.4 Teacher collaboration

The Health Promotion with Schools Framework states that for schools to incorporate effective health promotion, programs should involve students, staff and parents in a team‐based approach.^27^ Teacher collaboration was a strong facilitator to adoption in primary schools, drawing on the interpersonal aspects of the ecological model, through the creation of a supportive network.^28^ This collaboration overcame the impact of various program implementation barriers in primary schools.

‘*That's been another good thing about having [another Stage 3 RAA teacher] as a buddy, because then we were able to bounce ideas off each other. And if she implemented something, or had a problem, we would try and work through those things together*.’ – Primary teacher

Similarly, teacher collaboration was a reason for program continuance in a primary school movement integration intervention called ‘TAKE10!’^41^; brainstorming ideas and sharing resources assisted teachers in incorporating the TAKE10! program into their weekly schedules.^41^ Collaboration between teachers was less apparent in secondary schools.

### 3.5 Cross‐curricular adoption

Generally, in primary schools, students belong to one class group and have one teacher with whom they spend most of the school day, hence implementation of RAA was flexible to that teacher's schedule. Comparatively, in secondary schools, students have different classes and teachers throughout each school day, consequently high exposure to the intervention was reliant on cross‐faculty adoption. RAA seemed to favour the primary school structure; secondary teachers stated:

‘*But it would be effective if high school teachers had their own class. Because they [the students] are moving all the time and you've got so much content to get through, some days you just don't have time to do it [RAA] because you've just got to sit down and get straight on with your work*.’ – Secondary RAA coordinator

In secondary schools, RAA coordinators and their Personal Development Health and Physical Education faculty were responsible for recruiting teachers to implement RAA, however failed to gain cross‐curricular support for the program, suggesting the need for stronger principal and school executive support.

‘*[we] had no other faculties want to buy‐in because of the extra workload… I really pushed it…however, unfortunately, didn't really get any to buy‐in*’ – Secondary RAA coordinator

The Health Promotion with Schools Framework emphasises the need to incorporate the delivery of effective health promotion within the school ethos and a broader school approach,^27^ this highlights the need to ensure that programs are designed to be sustainable for teacher implementation,^27^ particularly in secondary schools.

### 3.6 Theme 2: Teacher development and resources

Supporting teachers to both understand the benefit of movement integration programs and increasing their skills to implement such interventions was a strong recommendation from both primary and secondary school teachers. Their feedback shed light on the influence of the intrapersonal factors of the ecological model for teachers, suggesting that each teacher's skills, attitudes and beliefs toward PA impacted their confidence in adopting the intervention, as also found by Webster and Colleagues.^21^

The Health Promotion with Schools Framework recommends training for teachers on specific health issues, providing information to parents and the school community, and identifying resources and expertise to ensure sustainable outcomes (eg, organisational support, adequate resources, relevant skills and knowledge).^27^

#### 3.6.1 Perceived benefits

As seen in other studies,^14^, ^21^, ^22^ teachers highlighted the need to demonstrate the benefits of active break interventions beyond the impact of PA on health, and promoting the benefit on school‐related outcomes such as cognition and behaviour, to motivate schools and teachers to adopt.

When referring to a PA intervention they had previously tried to implement, secondary teachers suggested:

‘…*teachers just thought of it as interruption to learning maths, [they say] “why am I doing energizer activities? That's your job. You're a PE teacher.” They don't see that embedding that cross curriculum is beneficial*.’ – Secondary RAA Coordinator

#### 3.6.2 Workout resources

Both primary and secondary school teachers found the RAA workout resources were a great facilitator for incorporating PA, suggesting they offered variety, enhanced student engagement and were flexible to the needs of their classrooms depending on space, student behaviour and time.

‘*The resources they provided were excellent for teachers*.’ – Primary RAA coordinator

‘*The resources kind of did activities and things that we don't normally do in the classroom*.’ – Primary teacher

‘*[the teachers] followed the circuits, all those cards that came with it, they did try and alternate that either daily or weekly, to provide a bit of variety for the kids*.’ – Primary RAA Coordinator

In their systematic review, Michael and colleagues^36^ discovered that a key barrier to implementing movement integration in primary schools was a lack of ideas for activities, and teachers reported the need for predeveloped activity resources.

Previous studies have shown that the design and flexibility of movement integration activities is predictive of classroom teacher's movement integration.^21^ The RAA resources were adaptable to time:

‘*It was an excellent fitness activity… you could do it for as shorter time or as long time to suit your needs in the classroom*.’ – Primary RAA coordinator

Following the intervention period, primary teachers utilised the workout resources during periods of online learning due to the COVID‐19 lockdown.

‘*And it's been really good now having those resources and having the experience with the program, because we've actually adapted it to our online learning as well*.’ – Primary teacher

Further, secondary teachers utilised the resources for wet weather days and times when they were confined to a small space.

‘*It did [help overcome barriers to PA], well especially for the teachers, because it gave them more things to do within their classroom. Because if they are confined to a small space, those activities are really good … simple and easy to do. And because they were easy, most kids got engaged*.’ – Secondary RAA coordinator

#### 3.6.3 Confidence

Few secondary teachers reported using the activity resources, however those that did suggested they enhanced engagement, were useful for student leadership, and would be facilitating for non‐health‐oriented teachers if they were to adopt the program. Comparatively in their study of a movement integration intervention in secondary schools, Innerd and colleagues^40^ discovered a lack of confidence amongst teachers in delivering activities that required physical skill and planning.

‘*It was great to have some stuff to show them (senior students) when they ran it (RAA)…. And I'm guessing that would be good for teachers who aren't physical education teachers as well*.’ – Secondary teacher

One primary school teacher recommended creating videos to support the activity resources.

‘*I really liked the launch video where the two people actually did the activities and the kids could follow along with them… because sometimes that was a bit of a barrier, having to say the activity and then me myself, having to demonstrate*’ – Primary teacher

### 3.7 Theme 3: Engagement with RAA program design elements

The RAA platform, in some cases, increased engagement with the program, by providing visuals and motivating students. However, in upscaling RAA, the School Sport Unit developed a new online platform which was unable to keep up with the increased volume and demand of those utilising the website. According to teachers, technical difficulties made interaction with the platform time consuming, limited the ability of students and teachers to experience the platform to its fullest potential and impacted student engagement. Teachers also suggested that a lack of consistent access to technology was a barrier to implementation in secondary schools.

‘*Technology in our school is scarce, at best. We have four computer rooms that are booked out continuously throughout the year. As far as laptops and iPads go…there's maximum of five per faculty. So anything technology based is a killer*.’ – Secondary RAA Coordinator

#### 3.7.1 Curriculum blogs

The Health Promotion with Schools Framework emphasises the need to ensure health promoting interventions are integrated into the curriculum.^27^ However, teachers had difficulty incorporating the curriculum blogs into their lessons, both due to time constraints and the curriculum content not aligning with the lesson plan at the time. Few secondary teachers reported using the content blogs and stated that they did not facilitate cross‐curricular adoption.

‘*But it's really hard to cross paths, because you do not know what other curriculum areas are doing, even though you ask them to join in and say that this would be beneficial, which it was, unless they are studying that, sometimes it's hard for them to put it in*.’ – Secondary RAA Coordinator

Previous studies have provided conflicting evidence on whether active breaks or active lessons (incorporating PA into class content), are more effective in increasing PA and producing school‐related outcomes.^17^, ^42^

#### 3.7.2 The ‘race’ element

In a pilot study of a primary school classroom active break intervention, gamification proved to improve children's motivation and engagement in PA, demonstrating a 27% increase in the number of students participating in MVPA.^43^ RAA adopts a gamification method through the race element of the platform, which proved to be a facilitator to students' engagement with PA, particularly in the primary schools, where it motivated students and encouraged class cohesion. In a systematic review of school‐based PA interventions in both primary and secondary schools, motivational interventions were deemed crucial for PA engagement, particularly for females.^44^

‘*But once we got going, and we could see our class move along the map, they were very competitive and wanting to log their hours in… It was great, they loved seeing that visual*.’ – Primary teacher

‘*…our school is a bit competitive, so it really appealed to the teachers and the students*.’ – Primary teacher

Primary school teachers suggested that the number of milestones diminished the goal setting effect of the unlockable milestones.

‘*Because there were so many milestones… we didn't really have a goal to get to it. Every time we logged on, we hit another milestone. Less, maybe bigger milestones… so that it's a bit more of a build‐up, a bit more exciting*.’ – Primary teacher

Feedback from secondary school teachers demonstrated varied utilisation of the competition element of RAA.

‘*I showed my classes at least once a week, because my two classes were competing against each other very frequently. By the end of the term, they were like, “Who won? Who won? We want to know!” Yeah. Really exciting for them*’ – Secondary teacher

‘*I don't know how much the kids liked the race part of it. From what I discussed with them, I'm not sure they were really into that*.’ – Secondary teacher

#### 3.7.3 Student leadership

A key aspect of successful implementation of school‐based PA interventions is establishing a multi‐component leadership committee, including students who may have the role of identifying enjoyable activities and promoting PA in the school.^45^ In primary schools, the race element of RAA offered opportunities for students to adopt leadership roles, which was further motivating for students.

In one primary school, a teacher stated:

‘*I've got a student, in my class who became the leader… And he was the first one in our class to be like, “right, we need to do two runs before we start our fitness today so we can get those kms up”…he was really motivating*.’ – Primary teacher

In primary and secondary schools, teachers suggested that if they were to implement RAA in the future, they would emphasise a student‐led component, to lighten the burden of implementation on teachers and to further engage students. After speaking about the difficulties faced in engaging teachers, one secondary school pondered:

‘*…do I get a small team that really want to be a part of this, and build up that way with a senior student who can oversee it and run it?*’ – Secondary RAA Coordinator

#### 3.7.4 Class cohesion

Further to peer leadership, primary school teachers reported that the race and team element of RAA, facilitated new social interactions and support networks within and between classes, which further contributed to enjoyment. The Health Promotion with Schools Framework states that effective school health interventions contribute to children feeling connected to their school and the community.^27^ It appears RAA facilitated this component in primary schools, however a stronger whole‐of‐school approach in both primary and secondary schools, could enhance this aspect of the program.

‘*So it was nice that they kind of use that time to come closer to people that they're not normally working with, because they're at very different academic levels or something. A couple of them actually commented that they enjoyed doing that, because they were able to do it with different peers*.’ – Primary teacher

### 3.8 Physical activity levels

Teacher feedback provided insight into whether the program increased movement and PA across the school day. In the primary schools, teachers introduced RAA activities as active breaks and transition tools, suggesting it increased PA levels. Systematic reviews and meta‐analyses have demonstrated the efficacy of classroom movement integration interventions in improving PA levels in youth and contributing to schools meeting MVPA guidelines.^15^, ^16^, ^17^

Participating primary school teachers, on average, reported facilitating a RAA workout 3 to 5 times a week, which is typical of teacher‐led movement integration interventions.^20^ Comparatively, in secondary schools, only Personal Development Health and Physical Education teachers adopted the program, implementing it during practical lessons and at times, as active breaks in theory lessons. However, exposure to RAA and therefore the impact of the intervention was low due to an overall lack of cross‐curricular adoption.

One primary school teacher specifically highlighted the increased PA participation amongst girls.

‘*…our girls were more eager to participate as it went on. So those participation levels for girls were really good*.’ – Primary RAA coordinator

There was evidence for RAA encouraging involvement in PA among students.

‘*And even some students that were a little bit reluctant towards PA, became involved. So I thought that part was great*.’ – Primary teacher

Teachers also suggested that it increased the students' self‐awareness of PA levels and encouraged students to complete more PA outside of school.

‘*I just noticed that some of my students were becoming more aware of how much PA that they were doing. And then we would have a discussion and there were certainly some that were trying to complete more, even on the weekends…*’ – Primary teacher

In secondary schools, low exposure seemed to impact the benefits perceived by teachers.

‘*It was good, it did increase involvement, but not to the extent that it was extra beneficial for most of the kids*.’ – Secondary RAA Coordinator

The feedback from both primary and secondary school teachers demonstrated the potential of RAA to increase PA levels when implemented as intended.

### 3.9 Cardiorespiratory fitness

Changes in primary school students' fitness outcomes, from baseline to follow up, are depicted in Table 3. The mean difference between the intervention and control groups was statistically significant (−36.91 seconds [−63.14, −10.68], *P* = .006) and indicated a small intervention effect for primary school students (*d* = 0.33). Few studies have explored this outcome previously. A study of a 10‐month ‘active lesson’ intervention in students 9 to 10 years of age, showed improvements in aerobic fitness for the least fit students, however, no overall effects were found.^46^ Further, in a study of an 8‐week curriculum‐based PA intervention in 9‐ to 11‐year‐olds, no substantial group by time effects were found for aerobic fitness.^40^

**TABLE 3:** Students' changes in 1.6 km run times from baseline to follow up

Outcome (secs)^a^	Intervention			Control			I‐C adjusted mean difference (95% CI)^b^	Treatment × time	Effect size (Cohen's *d*)
	Baseline mean (95% CI)	Follow up mean (95% CI)	*P* value	Baseline mean (95% CI)	Follow up mean (95% CI)	*P* value		*P* value	
Primary students	698.77 (673.60, 723.91)	671.04 (648.11, 693.96)	**.000**	688.67 (657.98, 719.35)	697.86 (659.16, 736.56)	.524	−36.91 (−63.14, −10.68)	**.006**	0.33
Secondary students	879.72 (165.68, 1593.75)	842.70 (136.01, 1549.40)	**.000**	860.60 (538.99, 1182.20)	835.35 (513.76, 1156.95)	**.000**	−11.78 (−33.54, 10.00)	.289	0.09

*Note*: Boldface indicates statistical significance (*P* < .05).

^a^ Time taken to complete the 1.6 km fitness test.

^b^ Adjusted for school clustering.

### 3.10 Primary school teachers' accounts of an overall change in attitude toward pa, and the use of workout resources beyond raa, reveal the potential for cardiorespiratory fitness outcomes to be sustained beyond the 9‐week intervention period

Changes in fitness outcomes from baseline to follow up for secondary school students are also depicted in Table 3. The mean difference between the groups was not statistically significant (−11.78. [−33.54, 10.00], *P* = .289), contrary to other movement integration intervention results in the secondary school setting.^40^, ^47^ There were no statistically significant differences between changes in fitness test times for gender in either primary or secondary schools.

## 4 AN ECOLOGICAL LENS ON RACE AROUND AUSTRALIA

It is indicative from the findings, that contextual factors, specifically at the intrapersonal, interpersonal and institutional levels of the Ecological Model,^28^ impacted the feasibility and acceptability of RAA and further influenced the effectiveness of the intervention. Overall levels of PA and in‐school PA have been shown to decrease significantly from childhood to adolescence^38^ hence, the findings from the qualitative component of this study are valuable in understanding the possible contributors to this trend.

At the institutional level, the differences in the segmentation of the school day and the exposure of teachers to students, favoured primary schools in terms of the feasibility of the intervention. A whole‐of‐school approach, with greater involvement from principals and executives, was needed to ensure sufficient support for teachers in both settings. In the secondary setting, this was particularly needed to support cross‐curricular adoption. Lack of access to technology in secondary schools was also raised as a barrier to student engagement. Further, it was evident that teacher development, in the form of raising knowledge and awareness, upskilling and resource provision, was needed to increase teachers' understanding of the benefits of movement integration and to enhance self‐efficacy in delivering the activities.

At the interpersonal level of the ecological model, support and encouragement amongst peers, teachers and class groups, through student leadership and within school competition, were facilitators for engagement and implementation. These themes were less prominent in secondary schools; however, student leadership was utilised in some instances and teachers suggested that strengthening this aspect could be an effective approach going forward.

At the intrapersonal level, primary school teachers and students demonstrated greater enjoyment in the intervention than secondary schools, due to both the feasibility of the intervention and the attitude of teachers and students. Teachers' skills, attitudes and experiences with classroom PA, influenced their adoption of the intervention. Lower levels of motivation amongst secondary school students when compared to primary school students impacted the ability of secondary school teachers to engage students.

A multi‐level ecological approach is needed to ensure the intervention is feasible and sustainable in both school settings across the various domains of the ecological model.

## 5 LIMITATIONS

Nonrandomisation could be a limitation of this study. In comparison with RCTs, in nonrandomised trials, there is a greater chance that the differences between treatment groups will influence observed outcomes in ways unrelated to intervention exposure, as often, nonrandomised trials rely on enthusiastic participants to volunteer allocation to the intervention group.^48^

Poor response rates for the student surveys highlighted the need for assistance from researchers with completion. Further, due to high numbers of missing data, the Baseline Observed Carry Forward imputation method was utilised, whereby all students with missing follow up data were assigned their baseline value, and all follow up data from students that did not have baseline data, were removed from analysis. This method is labelled as conservative, and therefore can strongly underestimate the effects of an intervention.^31^ Ideal methods for treating missing data are highly debated.^31^

Lastly, this evaluation failed to measure a dose‐response effect for the intervention, and therefore it is difficult to make meaningful comparisons between exposure and outcome. Choosing an appropriate measure for PA in youth is complex.^49^ The study demonstrated the need to pilot tools and implement the selected tools with researcher supervision. We recommend using both cardiorespiratory fitness testing as a proxy‐indicator for PA, and accelerometers to provide evidence of the effect of the intervention on in‐ and out‐of‐school physical activity levels.

## 6 CONCLUSION

The current research was the first known to directly compare the feasibility and acceptability of a 9‐week movement integration intervention across primary and secondary school settings. This research extends on current evidence of the potential of school active‐break interventions to improve students' cardiorespiratory fitness, demonstrating the contribution of such interventions to increasing children PA levels during the school day. Future developments of PA interventions in schools should utilise a multi‐level ecological approach, addressing the key barriers to implementation, specific to each setting. Future implementation efforts should be directed toward the secondary school setting.

## FUNDING INFORMATION

This work was supported by the Prevention Research Support Program, funded by the New South Wales Ministry of Health. Contributions were also made by the New South Wales Department of Education School Sport Unit.

## CONFLICT OF INTEREST

All authors declare that they have no conflicts of interest.

## ETHICS STATEMENT

Ethics approvals were sought and gained from: the University of Wollongong Human Research Ethics Committee (Reference 2021/ETH00065) and the NSW State Education Research Applications Process (SERAP).

## PATIENT CONSENT

Through opt out consent, all participants have consented for this research to be published.

## Supporting information


**Figure S1**: Screenshot of Race Around Australia onlilne platform.

**Figure S2**: Screenshot of Race Around Australia virtual race.

## Data Availability

Quantitative data are available from the corresponding author on reasonable request. Data for this study is not openly available as participants did not provide informed consent for data sharing.
