# Supplementary figures and images for: Feasibility, acceptability and potential efficacy of a virtual physical activity program in primary and secondary schools in New South Wales, Australia: A quasi‐experimental study
Source: Health Promot J Austr. 2022 Sep 23;34(1):70–84. doi: 10.1002/hpja.662 (PMC10087342; doi:10.1002/hpja.662)

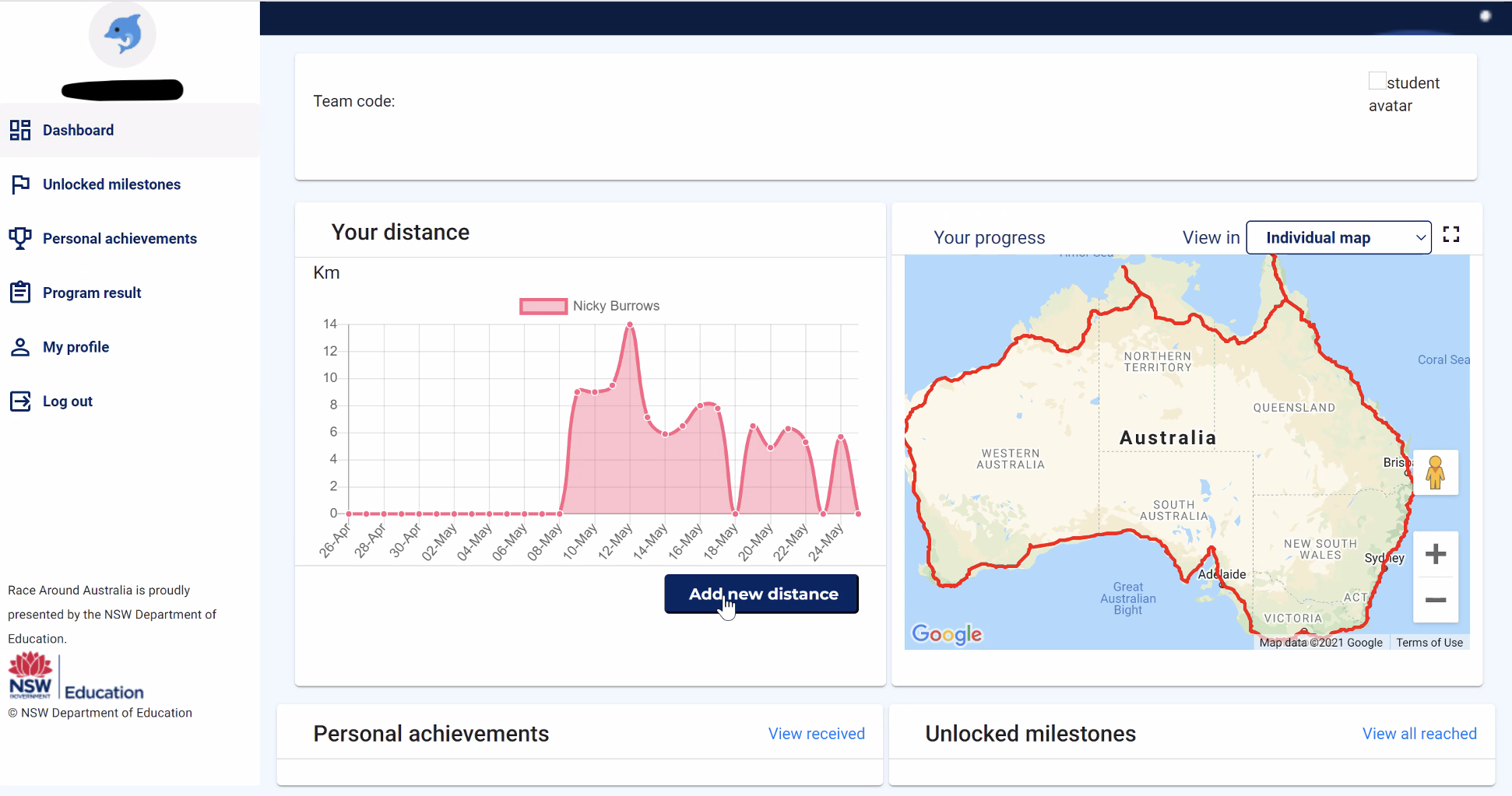

Supplement: Supplementary file 1 — Figure S1: Screenshot of Race Around Australia onlilne platform. [file HPJA-34-70-s001.png]

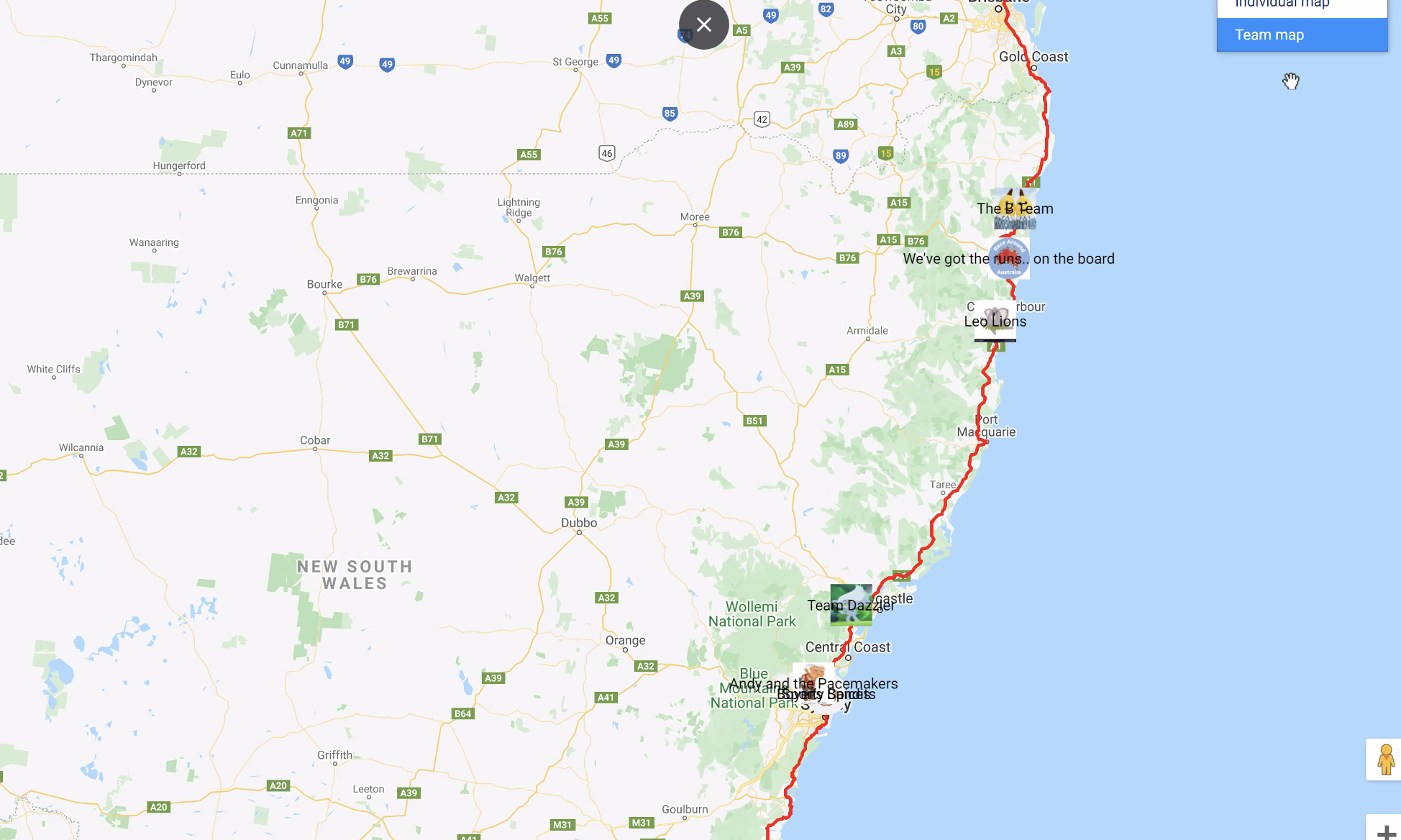

Supplement: Supplementary file 2 — Figure S2: Screenshot of Race Around Australia virtual race. [file HPJA-34-70-s002.png]
